# Design, creation, and use of the Test Us Bank (TUB) COVID-19 sample biorepository

**DOI:** 10.1186/s12879-024-10154-0

**Published:** 2024-11-12

**Authors:** John Broach, Chad Achenbach, Stephanie Behar, Laurel O’Connor, Seanan Tarrant, Julia Ferranto, Colton Wright, Paul Hartin, Taylor Orwig, Janvi Nanavati, Benedict Kalibala, Kelsey Woods, Bernadette Shaw, Julie Flahive, Bruce Barton, Nathaniel Hafer, Carly Herbert, Nisha Fahey, Laura Gibson, Karl Simin, Timothy Kowalik, Doyle V. Ward, Agha W. Mirza, Robert L. Murphy, Matthew Caputo, Bryan Buchholz, Heidi Fantasia, Ainat Koren, Lisa Marchand, Simisola Oludare, Felix Sogade, Dana Ritland, Cedrice Davis, Allen Grenier, Christi Baron, Ellie Brent, Jennifer Bacani McKenney, Nancy Elder, LeAnn Michaels, Laura Ferrara, Grant Theron, Zaida Palmer, Barcey Levy, Jeanette Daly, Kim Parang, Megan Schmidt, Denis Buxton, William Heetderks, Yukari C. Manabe, Apurv Soni, David McManus

**Affiliations:** 1 Department of Emergency Medicine, University of Massachusetts Chan Medical School, Worcester, MA USA; 2 Division of Infectious Diseases, Department of Medicine, Havey Institute for Global Health, Feinberg School of Medicine, Northwestern University, Chicago, Illinois 94305 USA; 3 Program in Digital Medicine, Department of Medicine, University of Massachusetts Chan Medical School, Worcester, MA USA; 4 Center for Clinical and Translational Science, University of Massachusetts, University of Massachusetts Chan Medical School, Worcester, MA USA; 5 Department of Population and Quantitative Health Sciences, University of Massachusetts Chan Medical School, Worcester, MA USA; 6 Department of Pediatrics, University of Massachusetts Chan Medical School, Worcester, MA USA; 7 Division of Infectious Diseases and Immunology, Department of Medicine, University of Massachusetts Chan Medical School, Worcester, MA USA; 8 Department of Molecular, Cell, and Cancer Biology, University of Massachusetts Chan Medical School, Worcester, MA USA; 9 Department of Microbiology and Physiological Systems, UMass Chan Medical School, Worcester, MA USA; 10 Division of Infectious Diseases, Department of Medicine, Johns Hopkins University School of Medicine, Baltimore, MD USA; 11 Department of Biomedical Engineering, University of Massachusetts Lowell, Lowell, MA USA; 12 Susan and Alan Solomont School of Nursing, University of Massachusetts Lowell, Lowell, MA USA; 13 Georgia Arrhythmia Consultants and Research Institute, Macon, GA USA; 14 Urban Family Practice, PC, Marietta, GA USA; 15 Department of Family Medicine and Community Health, University of Kansas Medical Center, Kansas, KA USA; 16 Fredonia Family Care, Fredonia, KA USA; 17 Oregon Rural Practice-based Research Network, Oregon Health and Science University, Portland, OR USA; 18 DSI-NRF Centre of Excellence for Biomedical Tuberculosis Research, South African Medical Research Council Centre for Tuberculosis Research, Division of Molecular Biology and Human Genetics, Stellenbosch University, Cape Town, MA South Africa; 19 Department of Family Medicine, University of Massachusetts Chan Medical School, Iowa, IA USA; 20 Department of Community and Behavioral Health, College of Public Health, University of Iowa, Iowa, IA USA; 21 National Heart, Lung, and Blood Institute, National Institutes of Health, Bethesda, MA USA; 22 Department of Emergency Medicine, National Heart, Lung, and Blood institute, NIH, via contract with Guidehousel, Bethesd, MD USA; 23 Division of Cardiology, Department of Medicine, University of Massachusetts Chan Medical School, Worcester, MA USA

## Abstract

Shortly after the first case of SARS-CoV-2 was diagnosed a public health emergency (PHE) was declared and a multi-agency response was initiated within the US federal government to create and propagate testing capacity. As part of this response, an unprecedented program designated Rapid Acceleration of Diagnostics (RADx) Tech was established by the National Institutes of Health (NIH) to facilitate the development of point-of-care tests for the COVID-19. The RADx Tech Clinical Studies Core (CSC), located at the University of Massachusetts Chan Medical School (UMass Chan), with partnering academic, private, and non-governmental organizations around the country, was tasked with developing clinical studies to support this work. This manuscript details development of a biorepository specifically focused on the collection and storage of samples designed for diagnostic platform development. It highlights the unified collection and annotation process that enabled gathering a diverse set of samples. This diversity encompasses the geography and backgrounds of the participants as well as sample characteristics such as variant type and RT-PCR cycle threshold (CT) value of the corresponding reference sample on a uniform clinical reference platform.

**Supplementary Information:**

The online version contains supplementary material available at 10.1186/s12879-024-10154-0

## Introduction

Since the emergence of severe acute respiratory syndrome coronavirus 2 (SARS-CoV-2), the need for reliable, accessible, and accurate testing for the presence of the virus and/or viral antigen has been a focal point of public health, clinical, and scientific response. A critical part of any public health response to an emerging infection is rapid and accurate identification of cases, which can be extremely challenging in the early and ongoing effort to reduce the impact of a novel pathogen. Indeed, the global community struggled in early 2020 to adequately identify cases of SAR-CoV-2 which hampered early containment and response programs [1–3].

Shortly after the first case of SARS-CoV-2 was diagnosed in the United States (U.S.), a public health emergency (PHE) was declared, and a multi-agency response was initiated within the federal government to create and propagate testing capacity for this novel pathogen [4]. As part of this response, an unprecedented program designated *Rapid Acceleration of Diagnostics* (RADx*) Tech* was established by the National Institute of Biomedical Imaging and Bioengineering (NIBIB) at the National Institutes of Health (NIH) and funded to facilitate the development of point-of-care (POC) tests for the coronavirus disease (COVID-19). The RADx Tech Clinical Studies Core (CSC), located at the University of Massachusetts Chan Medical School (UMass Chan), with partnering academic, private, and non-governmental organizations around the country, was tasked with developing clinical studies to support this work [5, 6]. As the virus evolved and surged in different parts of the U.S. and around the world, it was clear that development of effective testing relied on the ability of scientists and device manufacturers to obtain prospectively collected human body fluid samples of different types with which to develop, validate, and test their novel diagnostic platforms [7, 8]. The RADx CSC, under the clinical study name *Test Us*, completed research quickly, but were limited by needing to set up sample collection sites and clinical studies infrastructure in areas of greatest local transmission (i.e., during case surges) and to test assays across successive variant waves, specifically during times when many COVID-19 positive patients would be able to be recruited. However, this limited the ability to rapidly develop tests and be ready to respond to each successive wave of infection.

As recruitment for RADx tech studies continued, it became evident that to effectively carry out device trials, it was essential to gather prospectively collected biologic samples from participants, together with pertinent clinical information, including symptomatology at the time of collection. In most of the U.S., COVID-19 surges were occurring with only low-level transmission between these spikes. In addition, because the virus was mutating rapidly, it was recognized that samples specific to different variants might be desirable for diagnostics validation studies. Recruitment for validation studies was hindered during periods of lower prevalence due to difficulty recruiting enough patients with the virus, especially given the need for both symptomatic and asymptomatic individuals.

The literature describes several biobanks that were established in response to the COVID-19 pandemic, some include unique populations such as pediatrics, perinatal individuals and those with post-acute sequelae [9–11]. The UMass Chan Center for Clinical and Translational Studies (UMCCTS) established a collection of remnant clinic specimens (UMass Chan IRB Docket H00021250). However, none of these biobanks focused on the acquisition, organization, and distribution of respiratory samples for the primary purpose of evaluating and validating the performance of diagnostic tests for SARS-CoV-2 detection. The need for curated biorepositories to address this gap has also been recognized as a key step in preparation for emerging infectious diseases.

For this reason, the CSC, with approval from the NIBIB, created the Test Us Bank (TUB) biorepository in 2021 with the goal of prospectively collecting samples from both symptomatic and asymptomatic subjects who were either known to have COVID-19 or who were highly exposed and suspected to be currently infected at the time of collection. This manuscript details development of this biorepository specifically focused on the collection and storage of samples designed for diagnostic test development during infectious disease outbreaks and pandemic level response. It highlights the unified collection and annotation process that enabled gathering a diverse set of samples. This diversity encompasses the geography and backgrounds of the participants, (i.e., symptomatology of the patient at time of collection, vaccination status), as well as sample characteristics such as variant type, and Reverse Transcription Polymerase Chain Reaction (RT-PCR) cycle threshold value of the corresponding sample on a uniform clinical reference assay.

### Purpose

The purpose of the TUB project was to obtain, curate, and distribute a diverse collection of human body fluid samples paired with clinical information that may be used by investigators or developers for SARS-CoV-2 research and assay development. There are four primary sources of banked samples included in the TUB biorepository: samples primarily collected under the TUB protocol at one of the U.S.-based CSC participating sites, samples provided from partners at the Johns Hopkins Biobank, samples collected by the UMass Center for Clinical and Translational Science (UMCCTS) Biorepository and Tissue Bank, and those collected by partners at Stellenbosch University in South Africa. The TUB biorepository includes samples obtained from around our RADx Practice-Based Research Network (PBRN) partners and partner site network as detailed in this report. A unified catalog of all samples has been created and will be used for the tracking and allocation of all requests for biorepository samples. Samples collected for the bank include mid-turbinate (MT), anterior nares (AN), nasopharyngeal (NP) nasal swabs, saliva, venous blood, and capillary blood. Samples in the UMass Chan RADx TUB biorepository and those collected for the Johns Hopkins Biobank are all fully consented, prospectively collected samples with full demographic and metadata profiles (Supplement 1). Those samples from the UMCCTS Biorepository are single, waived consent, remnant samples which can be annotated upon request.

### Methods

Beginning in 2021, after approval from the NIBIB leadership and relevant NIH program officers, the CSC developed an operational plan to begin collecting samples from participants to support TUB. Recruitment was conducted in conjunction with diagnostic test validation studies or among patients who did not qualify to participate in validation testing protocols. Standardized protocols were developed for TUB and implemented by the CSC, who provided training and quality assurance to all sites. The UMass Chan institutional review board (IRB) approved the biorepository protocol (UMass Chan IRB Docket H00022475) and acted as the central IRB to which all other recruiting sites ceded. Initially, samples collected were nasopharyngeal and mid-turbinate nasal swabs, saliva samples, venous blood, and capillary blood. AN swabs were added after initiating TUB in October 2021. Swabs were stored in media or dry tubes to meet the needs of a variety of experiments. Our protocol allowed for participants to provide sample types of their choosing and the only requirement was that they provide at least one mid-turbinate nasal swab for testing by a highly sensitive RT-PCR assay, Roche Cobas 6800, to determine subject's infection status and at least one body fluid sample for the TUB repository. Throughout the development of the methods, harmonization of recruitment procedures and data collection was prioritized and was facilitated by a shared enrollment and participant interface system developed by collaborators at the University of California San Francisco (UCSF) known as Eureka (app version v.1.8.5–1.12) [12]. Recruitment at domestic RADx sites occurred between May 2021 and March 2022. In addition to collection sites in the RADx network in the U.S., a partnership was developed with Stellenbosch University in South Africa and recruitment of participants occurred there between May 2022 and April 2023.

### Study population

Initial inclusion criteria for participants providing samples to the biorepository were adults ages 18 or older willing to provide the requisite samples. Patients could be symptomatic or asymptomatic at the time of recruitment and there was no restriction based on whether a recent or past COVID-19 test had been performed or the results of that testing. Patients were recruited at each participating study site according to local opportunities available, targeting areas where COVID-19 testing was difficult to obtain for community members. Both inpatient and outpatient settings were included as possible recruitment sites and included those who had both mild symptoms as well as those who required hospitalization for more severe disease. After the first phase of collection, pediatric participants aged 6–17 years of age were invited to participate as well, with parental consent and self-assent.

### Sample collection procedures

**Fig. 1:**
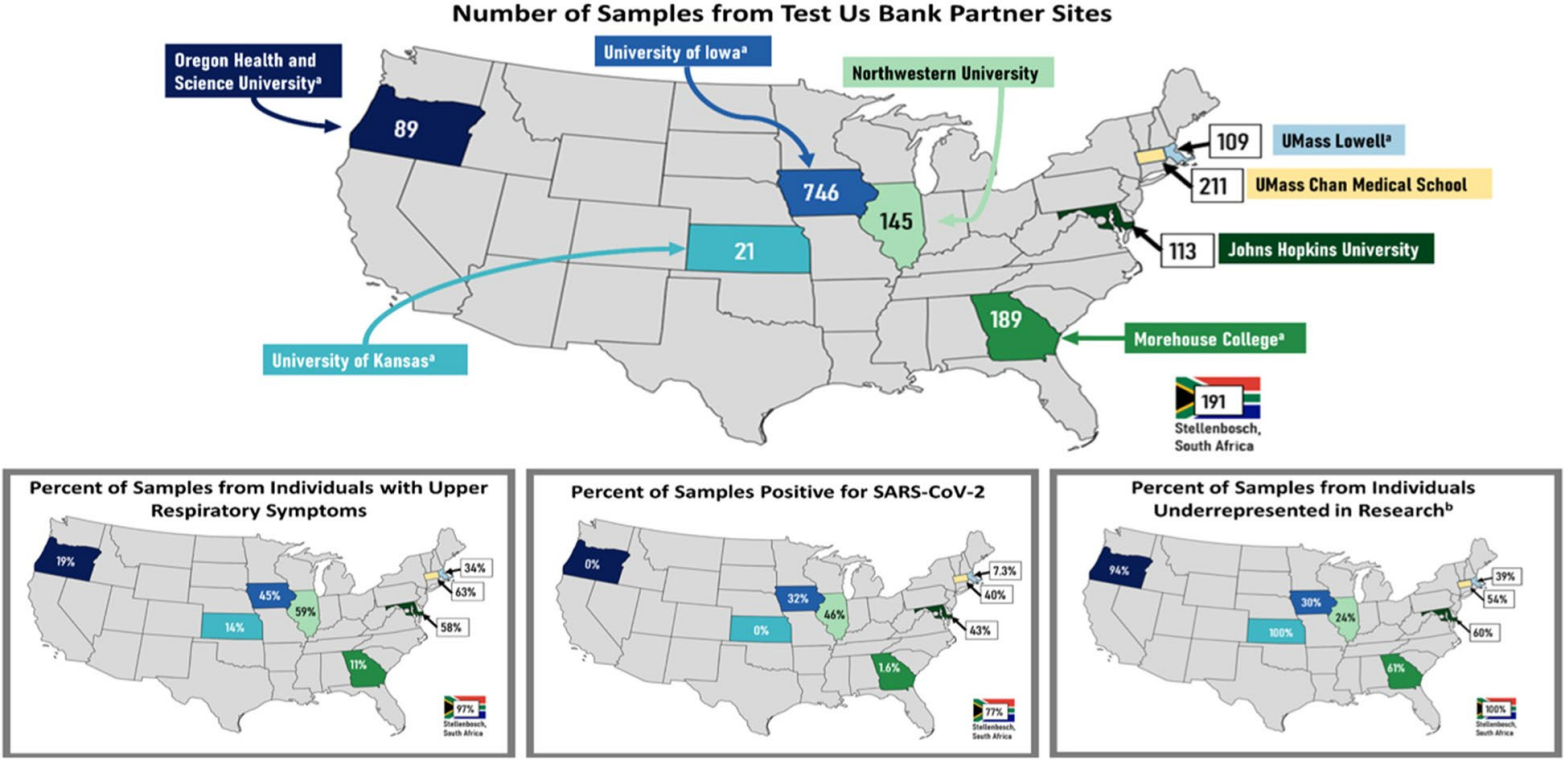
Test us bank partner sites and sample demographics. a Practice-based research network (PBRN) partner. b Underrepresented in research was defined as non-White, Hispanic, rural (defined as Rural Urban Commuting Area (RUCA) score 4–10), uninsured, or on Medicaid

All participants provided one MT swab sample for index COVID-19 testing and at least one, but up to four, additional sample(s) for the biorepository which could include: another MT swab sample, an AN swab sample, NP swab sample, a saliva sample, and/or a blood sample collected by venipuncture or fingerstick. The research samples provided were chosen by the participant at time of enrollment, through the eligibility survey delivered (Supplement 2) on the UCSF Eureka research platform. This enrollment process automatically assigned all participants with a six-digit unique participant identifier (Eureka ID). This platform also allowed the collection of standardized information from each patient including racial, ethnic, and linguistic background, COVID vaccination status, previous infections with COVID, as well as a survey of symptoms at the time of collection. Labels, each with a unique barcode ID were generated and assigned to each collected research sample. Samples were collected by a research nurse, physician, or research coordinator with specialized training. If provided, the NP sample was always collected first, followed by standard MT sample for index COVID-19 testing. Following the index COVID-19 sample collection, participants would give optional research samples, as applicable, in the following order: MT sample, AN sample, saliva sample, followed by a blood draw or fingerstick. At the time of collection, participant tracking forms were completed, listing the Eureka ID of the patient, barcode IDs of the samples collected, and the types of samples collected, including the time and date of collection (Fig. 1). AN, MT, and NP sample collection swabs were immediately placed in either dry tubes or tubes containing 5 mL of either universal or viral transport media. Samples were also immediately labeled with barcoded ID labels and individually placed in biohazard bags, and all samples collected from a single participant were placed together in a larger, collection biohazard bag, also labeled with a matching barcode ID label. These collection biohazard bags were temporarily stored and transported at -40 °C. These samples were brought to a lab at the end of the same day. For sites that did not have an accessible lab, samples were shipped overnight at -40 °C to UMass Chan, where they were promptly processed. Blood samples were bagged separately with barcode labels affixed to them and were transported separately in their own biohazard bags at ambient temperature for prompt processing. Blood samples were delivered to the lab and processed as soon as possible, but no later than the end of that same day. Once serum, plasma, and/or PBMCs were isolated, the samples were frozen at -80 °C. A chain of custody form was utilized through the web platform REDCap (v. 11.0.1), in which one form was created per participant, linked using the Eureka ID and sample barcode IDs. The chain of custody form listed research samples collected, and each time they were stored or transported in ice coolers, shipping boxes, or biorepository freezers. This ensured documentation of proper storage was provided for all samples from the moment of collection. While the storage and shipping protocols differed between sample types, as described above, all participating sites adhered to a common standard operating procedure.

### Sample processing and storage

The sample management process is summarized in Fig. 1. After sample collection from remote sites, MT, NP, AN, saliva, and dried blood spot samples were stored at –80 °C at their respective site prior to being shipped to UMass Chan Medical School. Since not all remote collection sites had biosafety facilities adequate for pipetting potentially infectious material, research samples were shipped on dry ice at -40 °C to UMass Chan Medical School for central processing in a Biosafety Level (BSL) 2 + lab. The remote sites received training for shipping Category B biological substances following International Air Transport Association (IATA) guidelines. The volume of dry ice used was determined by box size and number of samples shipped from remote collection sites to UMass Chan. Samples collected by UMass Chan were stored on dry ice at the time of collection and transported directly to a BSL 2 + lab, where the samples were handed off to biorepository staff. REDCap chain of custody logs were completed upon sample shipment to UMass Chan biorepository, denoting the change in transportation/storage location. At the UMass Chan biorepository, liquid samples (AN, MT, NP, and Saliva samples) were thawed and aliquoted into 0.5 mL aliquots and stored at -80 °C, and this procedure was cataloged for future access along with chain of custody documentation. A single aliquot of either swab media or saliva was used as a template for viral genomic sequencing. Venous whole blood was processed for plasma, serum, and/or peripheral blood mononuclear cells (PBMCs) within 24 h of collection using standard methods. After processing, plasma and serum were placed in freezers at -80ºC, while PBMCs are stored in liquid nitrogen freezers.

### Site selection

The RADx CSC onboarded several sites affiliated with the recruitment effort. These included other academic medical centers across the United States, mobile testing sites, as well as PBRNs and testing facilities across the United States. These sites were given the option to participate in recruitment for TUB in addition to any other ongoing clinical validation studies of COVID-19 diagnostics through the CSC. Participating sites are summarized in Fig. 1. This allowed our bank to obtain samples from a variety of geographic areas and to target recruitment in local areas of highest prevalence, in addition to adding to the diversity of the participants. Protocols were developed to ensure intact cold-chain logistics as well as chain of custody to ensure that samples were usable. Partnership with an international partner allowed further diversification of the samples with respect to variant type, and, in some cases, before certain variants were present in the U.S.

Each participating site utilized local recruitment methods best suited to their situation and opportunities to access participants. These varied widely but included partnering with local mass testing efforts, lists of patients known to have COVID-19, mobile testing sites, and many others. Each site also used available resources to increase diversity among participants and to ensure optimal community representation. Details about how sites accomplished this are presented as Supplement 3.

### Sample cataloging

Once received and processed, specimens were recorded using an OpenSpecimen (v. 8.0-) database to track inventory. Information from OpenSpecimen was integrated with sample information from the Eureka platform using SAS (v. 9.4) to develop a comprehensive sample catalog. Metadata and sequencing information, intended to allow for utilization of the samples for specific Emergency Use Authorization (EUA) and other regulatory claims, is attached to all samples enabling requestors to select samples known to be positive or negative for SARS-CoV-2 as well as identify matched samples across different sample types. The catalog report is maintained in real-time to reflect incoming and outgoing samples to accurately reflect the current availability of samples in the bank.

### Sample allocation

The purpose of the biorepository is to facilitate rapid validation of novel diagnostics; therefore, the biorepository developed a robust but expedited review process to consider requests for samples. Information about the Biobank is distributed to potential clients via announcements on the public website and at scientific meetings, and through electronic communications with companies and investigators.

Technology developers and academic investigators can request samples specifically to validate novel diagnostics, calibrate protocols across a variety of CT values and build evidence for claims regarding both symptomatic and asymptomatic home testing. Samples for the biorepository were collected in many cases by non-healthcare professionals and by patient self-collection, adding to the validity of the samples for home testing and over-the-counter device claims.

Investigators from all sectors are invited to request samples and/or data through the Test Us Bank Resource Allocation Committee (TUBRAC) by submitting an online request to the biorepository through a public-facing REDCap form (https://qmcsecure.ummsresearch.org/surveys/?s=77J7LHARXL). Requests are reviewed on a rolling basis and evaluated on the potential of the research being conducted to advance the science of COVID-19 testing, as well as to improve knowledge about COVID-19 and SARS-CoV-2.

The standardized electronic request form consists of questions about the nature of the proposed investigation and the quantity and characteristics of required specimens, provided by interested parties. Once an initial request is received, it is first reviewed by the lab and data management teams to ensure that the requested samples are available; the team’s coordinators subsequently correspond with the requestor for additional information if needed to determine availability.

When the inventory is confirmed, the request is adjudicated by a convened “Test Us Bank Resource Allocation Committee” (TUBRAC) which responds to all requests for the bank’s resources. The TUBRAC is comprised of stakeholders from each site that contributed samples to the bank and meets as needed along with members of the data and lab teams to review pending sample requests. The committee is guided by a standard operating procedure that delineates committee procedures, consensus requirements, and the hierarchy of allocation priority. Factors including the type of requestor, number and type of sample requests, and proportion of the requests with respect to available inventory. The TUBRAC votes in real time at each meeting to approve a sample request.

Once approved, a listing of the samples with complete metadata is provided to the requestor to ensure that the samples allocated will serve the purpose of the requester. A Tissue Transfer Agreement is executed between UMass Chan Medical School and the requesting party, and a highly monitored shipping strategy is executed to ensure that samples are appropriately handled during packing, transit, and receipt of samples.

## Results

One thousand six hundred twenty-three participants were recruited under the Test Us Bank protocol between May 2021 and March 2022 (Table 1). The biorepository includes samples from participants whose median age is 39 years old, with roughly an equal split between female and male participants (59 and 41 percent respectively). Although predominantly Caucasian, our population does include significant diversity with 25% of participants identifying as either a race other than white or as more than one race. The participants who provided samples reported a variety of COVID-19 symptoms at the time of enrollment, with the average number of reported symptoms among COVID-19 positive participants being 2.5 (SD = 2.0), and with “cough” and “runny nose” being the most commonly reported. The median time at enrollment from symptom onset was six days, with an interquartile range from 3–8 days. Reported symptomatology was higher among participants who tested positive for COVID-19. A significant majority (86%) of participants reported having had either one or two COVID-19 vaccinations, and there was no large difference between vaccination rates among participants who were positive or negative for COVID-19.

**Table 1:** TUB study participant characteristics^a^

Characteristic	Overall number of participants		SARS-CoV-2 PCR positive		SARS-CoV-2 PCR negative	
	(*n*=1814)		(*n*=561)		(*n*=1253)	
Demographics	n or median	% or IQR	n or median	% or IQR	n or median	% or IQR
Age at registration	39	(27, 56)	38	(29, 54)	40	(27, 56)
Sex: Female	1090	60	340	61	750	60
Race^b^						
White	1201	74	353	79	848	72
Black/African	158	9.7	27	6.0	131	11
Asian	89	5.5	12	2.7	77	6.6
Native American	4	0.25	2	0.45	2	0.17
Pacific Islander	8	0.49	2	0.45	6	0.51
Other	82	5.1	27	6.0	55	4.7
Unknown	23	1.4	9	2.0	14	1.2
More than one	58	3.6	15	3.4	43	3.7
Ethnicity^b^						
Yes: Mexican, Mexican American or Chicano	59	3.6	19	4.3	40	3.4
Yes: Puerto Rican	39	2.4	17	3.8	22	1.9
Yes: Cuban	1	0.06	0	0	1	0.09
Yes: Other or Mixed Hispanic, Latino or Spanish origin	92	5.7	28	6.3	64	5.4
Don't know	9	0.55	1	0.22	8	0.68
Prefer not to answer	8	0.49	0	0	8	0.68
Site						
Iowa	746	41	237	42	509	41
JHU	113	6.2	49	8.7	64	5.1
Kansas	21	1.2	0	0	21	1.7
Morehouse	189	10	3	0.53	186	15
NU	145	8.0	66	12	79	6.3
Oregon	89	4.9	0	0	89	7.1
Stellenbosch	191	11	114	20	77	6.2
UML	109	6.0	8	1.4	101	8.1
UMass Chan	211	12	84	15	127	10
COVID-19 Symptoms						
Scratchy throat^b^	0	0	0	0	0	0
Sore throat	105	5.8	65	12	40	3.2
Cough	469	26	320	57	149	12
Runny nose	464	26	281	50	183	15
Fever/chills	262	14	182	32	80	6.4
High temperature	67	3.7	55	9.8	12	0.96
Muscle aches	108	6.0	70	12	38	3.0
Nausea/vomiting/diarrhea	163	9.0	120	21	43	3.4
Shortness of breath	161	8.9	112	20	49	3.9
Unable to taste/smell	225	12	185	33	40	3.2
Red eyes^b^	0	0	0	0	0	0
Time from symptom onset (days)	6	(3, 8)	6	(4, 8)	5	(2, 10)
High blood pressure or hypertension	395	22	120	21	276	22
Diabetes	202	11	42	7.5	160	13
Coronary artery disease or angina	55	3.1	16	2.9	39	3.2
Heart attack	37	2.1	9	1.6	28	2.3
Congestive heart failure	21	1.2	8	1.5	13	1.1
Stroke or TIA	33	1.9	7	1.3	26	2.2
COPD	44	2.5	11	2.0	33	2.7
Asthma	129	7.1	41	7.3	88	7.0
Cancer undergoing active treatment	46	2.5	17	3.0	29	2.3
Immunodeficiency	25	1.4	12	2.2	13	1.0
Chronic HIV infection	15	0.83	5	0.89	10	0.80
Anemia or other blood disorder	114	6.3	29	5.2	85	6.8
Decreased kidney function or failure	32	1.8	14	2.5	18	1.4
CHD	0	0	0	0	0	0
CF	0	0	0	0	0	0
CLD BPD	0	0	0	0	0	0
Sickle cell anemia	2	0.11	0	0	2	0.16
Physical or mental condition(s) that limit ability to perform daily activities^b^	73	4.5	17	3.8	56	4.8
Deaf or have serious difficulty hearing^b^	7	9.6	2	12	5	8.9
Blind or have serious difficulty seeing^b^	9	12	2	12	7	13
Serious difficulty concentrating, remembering, or making decisions^b^	32	44	9	53	23	41
Serious difficulty walking or climbing stairs^b^	25	34	5	29	20	36
Difficulty dressing or bathing^b^	8	11	1	5.9	7	13
Difficulty doing errands alone^b^	27	37	8	47	19	34
Mental illness	397	25	134	31	263	23
Primary SARS-CoV-2 Vaccine Series (2 shots)	1504	83	480	86	1024	82
AstraZeneca	4	0.27	0	0	4	0.39
Janssen	182	12	92	19	90	8.8
Moderna	462	31	108	22	354	35
Novavax	1	0.07	0	0	1	0.10
Pfizer	834	55	277	58	557	54
Booster SARS-CoV-2 Vaccine^c^	379	25	121	25	258	25

^a^Patient Characteristics not available for CCTS Samples

^b^Not recorded for samples from Stellenbosch University

^c^Subset of SARS-CoV-2 vaccinated participants

One goal of our biorepository was to be a resource for developers of novel testing devices. For regulatory claims, symptomatology at the time of collection is critical and this information is presented in Table 2 with cross-reference of all COVID-19 positive patients by both symptomatology and vaccination status is presented in Table 3.

**Table 2:** Current biorepository sample types and aliquot catalog

Sample Type	All Negative			All Positive			Symptomatic Positive*b*			Asymptomatic Positive*b*		
	Samples*a*	Aliquots	Total Specimens	Samples*a*	Aliquots	Total Specimens	Samples*a*	Aliquots	Total Specimens	Samples*a*	Aliquots	Total Specimens
Anterior Nares swab	170	224	394	261	610	871	225	544	769	36	66	102
Mid-turbinate swab	784	1,489	2,273	366	827	1,193	318	734	1,052	48	93	141
Nasopharyngeal swab	540	654	1,194	8,516	19,628	28,144	151	427	578	20	37	57
PBMC		8	8		25	25		21	21		4	4
Plasma		817	817		525	525		453	453		72	72
Saliva	1,042	1,707	2,749	10,923	12,567	23,490	258	663	921	45	131	176
Venous blood	118	4	122	80		80	68		68	12		12

^*a*^Original samples provided by participant that have not yet been separated into aliquots

^b^CCTS samples do not include patient characteristics such as symptomatology

**Table 3:** Current specimen catalog stratified by vaccination status

Specimen Type	Symptomatic Positive (vaccinated)		Symptomatic Positive (unvaccinated)		Asymptomatic Positive (vaccinated)		Asymptomatic Positive (unvaccinated)	
	Samples^a^	Aliquots	Samples*a*	Aliquots	Samples^a^	Aliquots	Samples*	Aliquots
Anterior Nares	202	492	22	52	31	57	5	9
Mid-Turbinate	278	641	38	88	40	80	8	13
Nasopharyngeal	142	389	9	38	19	37	1	
PBMC		17		4		4		
Plasma		318		131		57		15
Saliva	228	589	28	70	37	112	8	19
Venous Blood	55		12		10		2	

^a^Original samples provided by participant that have not yet been separated into aliquots

All appropriate samples were processed by our biorepository team and split into aliquots to increase the number of samples available to the research community. For most sample types, between two and seven aliquots were obtained from each sample. All specimens are cataloged, and a summary of this catalog is available to requesters. The numbers of aliquots per sample are represented as Table 4 below.

**Table 4:** Average # of aliquots per sample type

	Samples	Aliquots	Average number of aliquots per sample^a^
Anterior Nares swab	451	893	3.5
Mid-turbinate swab	1180	2407	3.4
Nasopharyngeal swab	527	1165	3.6
Venous blood/Plasma	207	1405	6.8
Saliva	1202	2598	2.7
Serum		4	

^a^This is an average per sample/per participant. It is likely that the average is an underestimate of the actual number of aliquots per sample. This is for several reasons: 1) It is based on the number of aliquots that exist in the TUB Openspecimen data. That means if an aliquot was sent for sequencing, it would not contribute to the average for that particular sample/participant. 2) In addition, there are instances where only part of the original sample has been separated into aliquots. Therefore the # of aliquots per those samples is lower than expected

Samples included in our biorepository were also sequenced and variant data are presented in Tables 5 and 6. The preponderance of samples are Delta and Omicron variants as participant enrollment and sample collection began in 2021.

**Table 5:** Specimens sequenced

	All Positive	Delta	Omicron	Other Variants^b^	Not yet sequenced
# of participants^a^	6,612	1,988	1,760	2,816	22,352

^a^When duplicate samples were sequenced for a participant only one sample from each participant is represented above

^b^Other variants include the following clades: 19A, 19B, 20A, 20B, 20C, 20D, 20E, 20F, 20G, 20H, 20I, 20 J, 21B, 21C, 21D, 21F, 21G, and 21H

Sequenced samples were matched to TUB participants using the barcode through a crosslink between their OpenSpecimen barcode and their Eureka ID

**Table 6:** Specimens in freezers by variant

Sample Type	All Positive			Delta			Omicron			Not Yet Sequenced		
	Samples^a^	Aliquots	Total Specimens	Samples^a^	Aliquots	Total Specimens	Samples^a^	Aliquots	Total Specimens	Samples^a^	Aliquots	Total Specimens
Anterior Nares swab	261	560	821	98	240	338	40	180	220	108	154	262
Mid-turbinate swab	366	785	1151	184	428	612	69	180	249	124	215	339
Nasopharyngeal swab	8,516	19,628	28,144	98	2,468	2,566	39	2,941	2,980	7,541	12,442	19,983
Plasma	0	525	525		154	154	0	67	67	0	264	264
Saliva	10,923	12,567	23,490	138	4,015	4,153	96	2,648	2,744	9,439	4,854	14,293
Venous blood	80	0	80	20		20	12		12	46		46

^a^Original samples provided by participant that have not yet been separated into aliquots

**Fig. 2:**
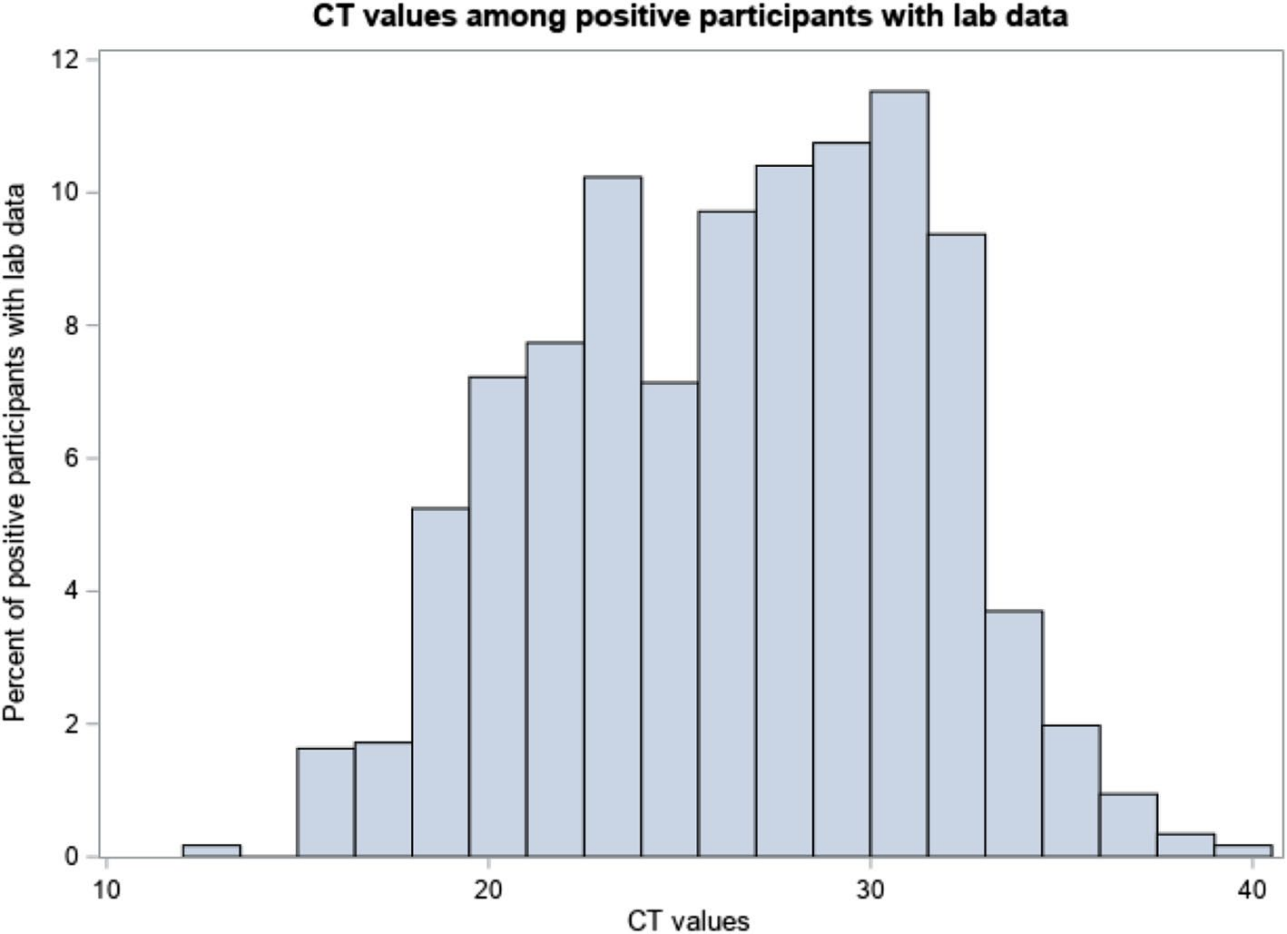
*CT values not available for CCTS and Stellenbosch samples

An important aspect of evaluating the performance of a novel SARS-CoV-2 diagnostic includes understanding the relative amount of virus in a sample being tested. The biorepository can provide partner researchers with data which signal the strength of the positivity of the reference sample associated with each aliquot. This data is available as cycle threshold (CT) values, as measured by the Roche Cobas 6800 RT-PCR assay for most samples. As both high and low CT value samples may be of use in different applications or for different research questions, Fig. 2 represents the range and frequency of available CT values in our samples in the TUB biorepository. The majority of CT values are less than 30 although there are a number of samples available with CT values as high as 40.

As the primary goal of TUB is to make samples available easily and efficiently to other researchers and diagnostics test developers, we report the number and types of samples that have been requested and provided as Table 7 below. At the time of the preparation of this manuscript, five sample requests have been received and shipped to partner researchers after review of the requests by the TUBRAC.

**Table 7:** Samples requested and sent by TUB as of February 14, 2023

Request	Samples Requested	Positive/Negative	Type of sample	Samples Sent	Mean CT	Date Shipped
1	30 positive and 30 negative samples of saliva (requiring samples of at least 600𝜇L) that also had MT or NP swabs with a CT value from one of the assays and pull the residual volume from the MT or NP swab that was tested if there is 300𝜇L or more left OR any untested MT or NP sample volume from the same subject	Neg	MT	45		5/27/2022
		Neg	Saliva	59		
		Pos	MT	46	27.5	
		Pos	Saliva	55	27.4	
2	A total of 40 matched saliva-swab samples tested with Roche Cobas: 20 positives all high CT & 20 negatives	Neg	MT	18		8/10/2022
		Neg	NP	1		
		Neg	Saliva	19		
		Pos	MT	18	32.3	
		Pos	NP	2	32.9	
		Pos	Saliva	20	32.3	
3	50 additional randomly selected positives samples	Neg	MT	21		10/17/2022
		Neg	NP	9		
		Neg	Saliva	46		
		Pos	MT	21	32.0	
		Pos	NP	12	31.3	
		Pos	Saliva	59	31.8	
4	20 positive and 20 negative dry swabs	Neg	ANT	15		10/27/2022
		Neg	MT	14		
		Neg	NP	4		
		Pos	ANT	18	26.1	
		Pos	MT	7	23.4	
		Pos	NP	1	31.9	
5	Saliva samples: 20 positive and 4 negative. Samples of 0.5-2 ml each	Neg	Saliva	15		10/31/2022
		Pos	Saliva	53	23.7	

## Discussion

The RADx Test Us Bank Biorepository (TUB) was established specifically to serve as a resource for researchers and technology developers working to design better SARS-CoV-2 diagnostics, especially rapid, at-home tests. The biorepository currently includes over 8,000 samples obtained from a demographically diverse set of participants both symptomatic and asymptomatic, multiple sample types, in a well-curated collection including molecular testing using an accepted reference lab assay and viral variants characterized by whole genome viral sequencing. Both positive and negative samples collected throughout the pandemic are included. Metadata for each sample includes symptomatology of the patient at time of collection, vaccination status, variant type, CT value of the corresponding reference sample, and demographics. The RADx TUB samples are currently stored in the UMass CCTS Biorepository and available through request (above, Sample Allocation).

The TUB biorepository was built to address developer needs for samples of different types, from participants with a variety of backgrounds and obtained in different individual and community contexts are necessary to truly support and fully understand the performance of novel testing platforms. Critically, it also includes samples from participants who were both symptomatic and asymptomatic with COVID, who had a wide variety of symptoms at the time of collection. The biobank also includes samples from individuals with respiratory symptoms but who tested negative for SARS-CoV-2 on PCR testing. This allows diagnostic technology developers to work with samples that support both symptomatic and asymptomatic regulatory claims and ensures that a representative cohort is included in their regulatory studies. The availability of our samples to the scientific community, and the publicly available “catalog” of samples is another way in which our effort was specifically designed to provide easy and transparent access to the samples as device researchers move quickly to develop assays.

In addition to its intrinsic value to researchers and technology developers (both those in the RADx program and from outside the program), the TUB serves as a model for creation of future biorepositories focused on support of developing novel diagnostics for other infectious diseases. As new threats emerge, the process used by our RADx CSC TUB team may serve as a template for how samples in future pandemics might be quickly made available for the critical public health task of developing reliable tests for emerging infectious diseases.

The primary purpose of this biorepository is to provide samples for novel diagnostics’ testing across different stages of the technology’s development lifecycle, including post-market experiments. Nevertheless, we envision several possible additional uses. The biobank has extensive metadata and captures sample collection over a wide range of the pandemic period, which can be useful for understanding natural progression of the pandemic and its impact based on participant reported characteristics. These data can facilitate an investigation of trends in infection status with respect to vaccination status, symptomatology, variant and subtype of the virus, demographics, and more. In addition to use for device development, we envision that samples from our biorepository could be used for a variety of other important purposes. Among these are investigations as to the prevalence of different variants by location and the progression of variant dominance over location and time as the pandemic evolved from its early stages through 2022. In addition, because variant data is available in this biorepository associated with symptomatology, viral tropism and associated symptoms over time may be of interest. These questions were and continue to be important throughout the pandemic. Public health officials and clinicians struggled to provide advice on what symptoms were likely to indicate presence of COVID-19. Similarly developed biorepositories in future pandemics could provide a source of ongoing data. These data could help determine which symptoms are associated with a novel infection and how these symptoms change with pathogen evolution over time and at different points in the clinical course of disease.

These data are available for researchers and other interested stakeholders’ use through the NIH RADx Data Hub [https://radx-hub.nih.gov/]. NIH RADx Data Hub is an effort coordinated by the NIH Office of the Director to democratize access to de-identified data produced by the NIH RADx program. Our clinical studies core is contributing data from the biorepository as well as a myriad of other studies that vary in time period and study design. The data from NIH RADx Data Hub can be requested by creating or using an existing Login.gov account.

### Limitations

The Test Us Bank biorepository, while large and consisting of several different sample types, nonetheless has several limitations. First, the samples were not collected in a fashion that was blinded to infection status. Some samples were collected from individuals with known disease, and this may affect the utility for testing claims. Despite significant efforts to include diverse populations in our protocol, racial and ethnic diversity was limited. Samples from both symptomatic and asymptomatic patients were collected and included in the biorepository. This was important to ensure that future use could include investigation of the performance of novel devices on patients that may have covid but be asymptomatic at the time of sample collection. However, this does introduce the possibility that some asymptomatic participants, particularly those with high-risk exposure to COVID, would later go on to develop infection that may not have been detectable in their initial sample. Additionally, among symptomatic patients that were negative for COVID-19, it is possible and even likely that they may have had another viral illness. While we acknowledge this, it is nonetheless important that they are included, particularly to ensure a cohort of samples that would require novel devices to distinguish between COVID-19 infection and other pathogens. As a cross-sectional analysis, long-term follow up of participants was not included and this may limit the utility of the data for research areas such as investigation into the long-term effects of COVID-19 or longitudinal changes in SARS-CoV-2, infectivity, or immune response within individuals. We also note that in future biorepository development, follow up of participants with data collection about their clinical course might create additional utility for investigators wishing to understand the predictive value of initial test results and patient characteristics on clinical outcome. Future directions could also include use of other measures of viral load in addition to Cycle Threshold such as a number of viral RNA copies per milliliter as an alternate or second measure of viral density in a sample [13]. It is also noted that use of the TUB has been more limited than desired. We speculate that this is partially due to the preference of many participants for providing saliva samples which were used less by test developers, especially in the creation of lateral flow assay devices. It is also possible that development of the TUB earlier in the pandemic would have contributed to its greater utilization when device development was most active. We feel that, although the TUB will remain a resource for future investigators, early development of similar biorepositories should be considered as early as possible in future epidemics and pandemics to maximize their utility to the scientific community.

## Conclusion

Our RADx Tech Test Us Bank Biorepository was built with the understanding that a contemporaneous pool of samples, collected through various phases of the pandemic, would be of value to researchers as they try to better understand how to test for COVID-19. By successfully incorporating numerous sites around the U.S. and internationally, the TUB biorepository was able to collect a large number of specimens and has made them widely available for use by other investigators. This distributed collection model as well as our focus on deep understanding of the participant and virus characteristics associated with each sample provides a model for successful biobanking during a pandemic. Although the recent utilization of the described biorepository has been more limited than desired, it is the hope of the authors that the approach, with a focus on collecting samples in such a way as to make them most useful to novel device developers as well as the academic community may serve as a model for future work in the creation of biorepositories.

## Supplementary Information

Supplementary Material 1.

## Data Availability

The data generated during the current study are available through the NIH RADx Data Hub (https://radx-hub.nih.gov/). Additionally, investigators may request samples and/or data through the Test Us Bank Resource Allocation Committee (TUBRAC) by submitting an online request to the biorepository through REDCap at https://qmcsecure.ummsresearch.org/surveys/?s=77J7LHARXL.
