# Supplementary material for: Design, creation, and use of the Test Us Bank (TUB) COVID-19 sample biorepository
Source: BMC Infect Dis. 2024 Nov 12;24:1282. doi: 10.1186/s12879-024-10154-0 (PMC11556042; doi:10.1186/s12879-024-10154-0)
Supplement: Supplementary file 1 — Supplementary Material 1. [file 12879_2024_10154_MOESM1_ESM.docx]

**Supplemental Material**

**Supplement 1 - Metadata Survey Instruments**

*Demographics*

1. What is your height (in feet and inches)?

__feet ___inches

2. What is your weight (in pounds)?

_____lbs.

3. What sex were you assigned at birth?

Male

Female

Prefer not to answer

4. What best describes your gender identity? (check all that apply)

Man

Woman

Non-binary

Transgender

None of these describe me, and I’d like to consider additional options

Prefer not to answer

[If ‘non-binary,’ ‘transgender,’ or ‘none of these describe me and I’d like to consider additional options’ selected]

Are any of these a closer description to your gender identity?

Trans man/Transgender Man/FTM

Trans woman/Transgender Woman/MTF

Genderqueer

Genderfluid

Gender variant

Questioning or unsure of your identity

None of these describe me, and I want to specify

5. What is your racial background? (Check all that apply)

White

Black or African American

American Indian or Alaska Native

Asian (including South Asian and Asian Indian)

Native Hawaiian or Pacific Islander

Some other race

I don’t know

[If “Asian” is selected]

What is your Asian background?

Chinese

Filipino

Asian Indian

Japanese

Korean

Vietnamese

Other Asian or Mix

[If “Native Hawaiian or Pacific Islander is selected]

What is your Pacific Islander background?

Native Hawaiian

Samoan

Guamanian or Chamorro

Other Pacific Islander or Mix

6. Are you of Hispanic, Latino, or Spanish origin or ancestry? (One or more categories may be selected)

No

Yes, Mexican, Mexican American, Chicano

Yes, Puerto Rican

Yes, Cuban

Yes, other or Mixed Hispanic, Latino, or Spanish origin

7. We would like to know about what you do – are you working now, looking for work, retired, keeping house, a student, or what?

Working now

Only temporarily laid off, sick leave, or maternity leave

Looking for work, unemployed

Retired

Disabled, permanently or temporarily

Keeping house

Student

Other (specify)

Please specify about what you do.

Are you currently enrolled in school full time (or on a regularly scheduled break, such as winter, spring, or summer break)? [Yes/No/Don’t know]

8. What is the highest grade or level of school you have completed or the highest degree you have received?

Did not finish high school

High School Graduate

GED or Equivalent

Some College, No Degree

Associate Degree: Occupational, Technical, or Vocational Program

Bachelor’s Degree (Example: BS, BA, AB, BBA)

Master’s Degree (Example: MA, MS, MEng, MEd, MBA)

Professional School Degree (Example: MD, DDS, DVM, JD)

Doctoral Degree (Example: PhD, EdD)

Prefer not to answer

Don’t know

[If “Did not finish high school” is selected]

Please choose the highest level of education you have completed.

Never attended/Kindergarten only

2^nd^ Grade

3^rd^ Grade

4^th^ Grade

5^th^ Grade

6^th^ Grade

7^th^ Grade

8^th^ Grade

9^th^ Grade

10^th^ Grade

11^th^ Grade

12^th^ Grade, no diploma

9. Are you covered by any of the following types of health insurance or health coverage plan? [Covered/Not Covered/Not Sure]

a. Inurance through a current or former employer or union (of yours or another family member’s). This would include extension of employer-based insurance after you left a job (i.e. coverage under COBRA

b. Insurance purchased directly from an insurance company (by you or another family member). This would include coverage purchased through an exchange or marketplace, such as HealthCare.gov.

c. Medicare for people 65 and older, or people with certain disabilities.

d. Medicaid, Medical Assistance (MA), the Children’s Health Insurance Program (CHIP), or any kind of state or government-sponsored assistance plan based on income or a disability. Sometimes different states have specific names for the Medicaid plans (for example MassHealth).

e. TRICARE, Veterans Affairs (VA) healthcare, or other military health care or health insurance

f. Indian Health Service

g. Any other type of health insurance coverage or health coverage plan not listed in previous questions

[If “covered” is not selected for any items above]

h. Does this mean you currently have no health insurance or health coverage plan? In answering this question, please exclude plans that pay for only one type of service (such as, nursing home care, accidents, family planning, or dental care) and plans that only provide extra cash when hospitalized.

I do NOT have health insurance

I HAVE some kind of health insurance

[If this “I HAVE some kind of health insurance” is selected]

What type of health insurance do you have?

10. How confident are you filling out medical forms by yourself? [Extremely/Quite a bit/Somewhat/A little bit/Not at all]

*Tobacco History*

1. Do you use any tobacco/nicotine products (including cigarettes, cigars, e-cigarettes, etc.)? [this question is optional] [Yes/No]

[If “yes” is selected]

About how much do you smoke cigarettes? [Every day/Some days/Not at all/Don’t know/Prefer not to answer]

About how much do you use e-cigarettes? [Every day/Some days/Not at all/Don’t know/Prefer not to answer]

About how much do you use cigars/cigarillos? [Every day/Some days/Not at all/Don’t know/Prefer not to answer]

About how much do you use a pipe filled with tobacco? [Every day/Some days/Not at all/Don’t know/Prefer not to answer]

About how much do you smoke hookah? [Every day/Some days/Not at all/Don’t know/Prefer not to answer]

About how much do you use smokeless tobacco? [Every day/Some days/Not at all/Don’t know/Prefer not to answer]

About how much do you use dissolvable tobacco? [Every day/Some days/Not at all/Don’t know/Prefer not to answer]

*Alcohol Use*

1. How often did you have a drink containing alcohol in the past year? [this question is optional]

Never

Monthly or less

Two to four times a month

Two to three times per week

Four or more times per week

2. How many drinks containing alcohol did you have on a typical day when you were drinking in the past year? [this question is optional]

1 or 2 drinks

3 or 4 drinks

5 or 6 drinks

7 to 9 drinks

10 or more drinks

3. How often did you have six or more drinks on one occasion in the past year? [this question is optional]

Never

Less than monthly

Monthly

Weekly

Daily or almost daily

*COVID-19 Experience*

1. Have you ever been tested for the novel coronavirus, the virus that causes COVID-19 (either a test to detect the virus for active infection or the antibody to detect past infection)?

Yes

No

Other

[If yes to the above question]

Was it a test for active infection (virus) or past infection (antibody to the virus)?

Test for active infection (virus)

Test for past infection (antibody to the virus)

I had both kinds of tests

I don’t know

[If active infection is selected]

About how many weeks ago was your last test for active COVID-19 infection (virus)?

Do you know the result of your last test for active COVID-19 infection (virus)?

Yes, I was positive (the novel coronavirus WAS detected)

Yes, I was negative (the novel coronavirus was NOT detected)

Yes, the test was inconclusive (could not tell whether the virus was present or not)

No, not yet

[If past infection is selected]

About how many weeks ago was your last test for past infection (antibody to the COVID-19 virus)? Put 0 if this week

___ weeks ago

Do you know the result of your last test for past infection (antibody to the COVID-19 virus)?

Yes, I was positive (antibody to COVID-19 WAS detected suggesting past exposure)

Yes, I was negative (antibody to COVID-19 was NOT detected suggesting NO past exposure)

Yes, the test was inconclusive (could not tell whether the antibody was present)

No, not yet

2. In the past two weeks, have you been in close contact (within 6 feet for more than 15 minutes) with anyone who recently (within 1 week of your exposure) tested positive for COVID-19 virus?

Yes

No

Other

[If yes is selected]

How many days ago were you exposed to someone who recently tested positive for the COVID-19 virus?

3. In the past 48 hours have you had any of the following? (check all that apply)

A sore throat

A cough

A runny nose

Congestion

Symptoms of fever or chills

A temperature greater than 100.4ºF or 38.0ºC

Muscle or body aches (worse than usual if you have baseline muscle aches)

Nausea, vomiting, or diarrhea

Shortness of breath

Difficulty breathing

Unable to taste or smell

Fatigue

Headache

None of the above

[If symptoms are selected]

Did you seek medical care for these symptoms?

Yes

No

If you have symptoms, how many days ago did the first symptom start?

4. IN THE PAST 48 HOURS, has anyone (other than you) in your household had any of those symptoms? [Yes/No]

5. IN THE PAST 48 HOURS, approximately how many people outside of your household did you interact with while they were within 6 feet? (“Interact” is loosely defined as talking, touching, or just being within 6 ft of someone for longer than 1 or 2 minutes)

6. Approximately how many of those people were wearing masks, or were behind a shield?

7. Have you ever received a COVID-19 (SARS-COV-2) vaccine? [Yes/No/I don’t know]

[If yes]

How many vaccine doses have you received?

[for each dose indicated]

What was the date of your [first, second, third] COVID-19 vaccine (OK to guess if unsure)?

Which company’s COVID-19 vaccine did you receive?

AstraZeneca

Janssen (Johnson & Johnson)

Moderna

Novavax

Pfizer

Other, Specify

I don’t know

8. Have you previously received any of the following treatments for COVID-19? (check all that apply)

Dexamethasone

Remdesivir (Veklury)

Casirivimab & Imdevimab (REGN-COV2)

Bamlanivimab (LY-COV555)

Monoclonal antibodies (antibodies made in a laboratory)

Convalescent plasma (antibodies collected from people)

Other therapy specifically treating COVID-19

I have received a treatment for COVID-19 but I do not know the name

I have never received a treatment for COVID-19

9. Are you currently receiving any of the following treatments for COVID-19? (check all that apply)

Dexamethasone

Remdesivir (Veklury)

Casirivimab & Imdevimab (REGN-COV2)

Bamlanivimab (LY-COV555)

Monoclonal antibodies (antibodies made in a laboratory)

Convalescent plasma (antibodies collected from people)

Other therapy specifically treating COVID-19

I am currently receiving a treatment for COVID-19 but I do not know the name

I am NOT currently receiving a therapy for COVID-19

*Medical Conditions*

1. Have you ever been told by a doctor or nurse that you have, or have been treated for, any of the following conditions (in the past or currently)? [Yes/No/Don’t know, unless otherwise indicated]

High blood pressure or hypertension (except that occurred during pregnancy and did not last after pregnancy)

Diabetes (do not include pre-diabetes)

Coronary artery disease (blockages in your heart vessels) or angina (chest pain)

A heart attack (myocardial infarction)

Congestive Heart failure (CHF, Heart Failure)

Stroke or TIA (Transient Ischemic Attack or Mini-Stroke)

COPD (emphysema, chronic bronchitis, obstructive pulmonary disease

Asthma, to the point that you use inhalers daily or have been to the hospital for your asthma

Cancer (including leukemia or lymphoma) undergoing active treatment

Immunodeficiency (NOT including HIV)

Chronic HIV infection

Anemia or other blood disorder (do not include leukemia or lymphoma)

Sickle cell anemia

Decreased kidney (renal) function or kidney (renal) failure

Yes, but not on dialysis

Yes and on dialysis

Yes and I’ve had a kidney transplant and my kidney function is now normal

No

2. Do you have any serious physical or mental conditions that limit your ability to perform daily activities? [Yes/No] [this question is optional]

[If yes is selected]

a. Are you deaf or do you have serious difficulty hearing? [Yes/No]

b. Are you blind or do you have serious difficulty seeing, even when wearing glasses? [Yes/No]

c. Because of a physical, mental, or emotional condition, do you have serious difficulty concentrating, remembering, or making decisions? [Yes/No]

d. Do you have serious difficulty walking or climbing stairs? [Yes/No]

e. Do you have difficulty dressing or bathing? [Yes/No]

f. Because of a physical, mental, or emotional condition, do you have difficulty doing errands alone such as visiting a doctor’s office or shopping? [Yes/No]

3. Have you ever been told by a physician, psychologist, or healthcare provider that you have any mental illness (for example depression, anxiety, bipolar, schizophrenia, or other)? [this question is optional]

4. Are you currently pregnant? [Yes/No/Don’t Know/Prefer not to answer] [this question is optional]

5. What medications are you currently taking?

Medication:

Strength:

How often do you take your medication?

*Follow-Up Survey Questions* *[Completed 2 weeks after sample collection]*

1. Since enrollment, have you had any of the following symptoms?

Sore throat

A cough (worse than usual if you have a baseline cough)

A runny nose

Congestion

Symptoms of fever or chills

A temperature greater than 100.4ºF or 38.0ºC

Muscle or body aches (worse than usual if you have baseline muscle aches)

Nausea, vomiting, or diarrhea

Shortness of breath

Difficulty breathing

Unable to taste or smell

Fatigue

Headache

None of the above

2. Did you test positive for COVID-19 after testing negative in the Test Us Bank study? [Yes/No]

**Supplement 2 - Eligibility Survey**

1. Are you 18 years or older? [Yes/No]

2. Are you willing to get tested for COVID-19 with a swab from the middle part of your nose (midturbinate)? [Yes/No]

3. Are you willing to answer questions about any symptoms and exposure to anyone with COVID-19 in the last two weeks? [Yes/No]

4. Are you willing to provide at least one of the samples below to be stored for future research on COVID-19? [Yes/No]

· Saliva sample

· Nasal swab (from the middle part of your nose)

· Nasopharyngeal swab (from the back part of your nose)

· Anterior Nares swab (from the front of your nose)

· Capillary blood from Fingerstick

· Venous blood (blood from your vein)

5. Which samples do you plan on providing in addition to your COVID-19 test? You can change your mind about which samples you are comfortable providing at any time (Check all that apply):

Saliva samples

Nasal swab (from the middle part of your nose)

Nasopharyngeal swab (from the back part of your nose)

Anterior Nares swab (from the front of your nose)

Capillary blood from Fingerstick

Venous blood (blood from your vein)

**Supplement 3 - Site Operations**

One unique aspect of our biorepository was the distributed nature of the collection effort and flexibility in our recruitment methods that allowed numerous sites to take advantage of local conditions that favored recruitment and access to patients. Detailed below are the common methods used for recruitment.

*Mass testing sites*

Throughout the pandemic, many states began large-scale testing efforts to provide easy access for individuals who were symptomatic or exposed to COVID-19. These testing sites were often overwhelmed with patients and RADx sites, particularly the University of Massachusetts Chan Medical School, formed partnerships with these mass testing sites to allow recruitment on site. In Massachusetts, the research team often recruited TUB participants by co-locating operations with Stop the Spread mass testing sites which were funded by the state of Massachusetts and operated by UMass Memorial Health.. Because our protocol included a PCR test with the result available to the participant in addition to the collection of research samples, we were able to offer participation in our study as an alternative at the testing event. For many RADx studies, large-scale testing events provided excellent recruitment opportunities.

*Known positive lists*

At a number of sites, agreements with large health systems, inpatient units, and community practices allowed access to daily lists of patients who had tested positive for COVID-19. At the UMass Chan, Northwestern University, the University of Iowa, and Stellenbosch sites, these lists were used to either identify patients who could then be contacted the day after their index positive test for recruitment. These patients were also offered the opportunity to refer close contacts for testing. Depending upon the site, a phone or email-based survey was used to screen for eligibility, and eligible qualifying patients were then either approached in their inpatient wards or asked to come to specially outfitted research rooms where samples could be safely collected without contaminating other patient care areas.

*Advertising*

Many sites used IRB-approved flyers and other advertising methods, and some sites also used specially-created organic and paid social media postings (NU, JHU, UMass Chan) with links to study websites and electronic eligibility surveys. Completed surveys were then reviewed by study staff, and the patients were contacted for follow-up recruitment.

*Targeting underrepresented populations through practice-based research networks (PBRNs)*

Across all sites, efforts were made to recruit from areas of known high case-load, as well as from specific populations, such as non-English speakers and persons of color, to ensure the most representative possible array of positive samples in the biorepository. The TUB biorepository worked. This biorepository was especially fortunate to work collaboratively with PBRN sites in Georgia, Kansas, Iowa, and Oregon as well as sites such as UMass Lowell that had access to clinics in both urban and rural settings. In a number of cases, these rural clinics required significant logistical consideration when collecting and transporting samples great distances.

For example, The University of Kansas Medical Center (UKMC) and its linked PBRN practice-based research network, Kansas Patients and Providers Engaged in Prevention Research (KPPEPR), engaged a rural practice in southeast Kansas to assist in recruitment and sample collection for TUB. This primary care practice, Fredonia Family Care, located in Fredonia, Kansas (almost 150 miles away from the main UKMC campus) serves a community of less than 2,500 individuals. Despite the size of the community, the practice was able to enroll over 20 individuals in TUB.

At the University of Iowa, Department of Family Medicine, the team accessed the Iowa Research Network (IRENE) to recruit participants for TUB at the University of Iowa Hospitals and Clinics (UIHC) Family Medicine clinic in Iowa City and West Broadway Clinic in Council Bluffs.

The Oregon Rural Practice-based Research Network (ORPRN) research team worked with One Community Health, a Federally Qualified Health Center (FQHC) in Hood River serving migrant farmworkers and North Central Public District, a tri-county health department in The Dalles both located in rural regions in Oregon. Both subsites agreed to recruit via patient referrals, advertisements, and by allowing ORPRN staff access to patients doing same-day COVID-19 testing.

At our partner sites affiliated with Morehouse University, specifically Urban Family Practice, located in the suburbs of Atlanta, recruitment was performed using the clinics database, as well as through community organizations.

*Mobile Sites*

Given the desire to recruit participants in a variety of locations, several sites used mobile testing and recruitment strategies. The aforementioned above-noted Council Bluffs, Iowa site required the Iowa-based research team to travel over 250 miles to the site for two three-day recruitment trips, which were pre-arranged with the local clinic so that clinic space could be utilized to enroll TUB participants. These events were advertised in advance and participants were scheduled to arrive at the clinic during the three-day period. Each night, samples were delivered to a nearby FedEx location to ensure the collected samples would arrive safely for processing at the biorepository.. In contrast, the UMass Lowell team used a mobile testing van, which was fully equipped to be able to safely recruit participants and collect samples from patients in a variety of locations across the greater Lowell, MA area.

*Diversity and Inclusion Efforts*

At many sites, such as the aforementioned PBRN sites, focus was placed on representation in our biorepository from communities of color and among participants whose primary language was not English. All sites were encouraged to focus recruitment efforts in locations with barriers to healthcare access based on social determinants of health, and this effort was exemplified by the availability of recruitment materials in multiple languages, across all sites.

*International Recruitment*

Through a longstanding collaboration between Northwestern University and Stellenbosch University, TUB was able to integrate samples from South Africa as well. The Stellenbosch Health System has very active research programs focused on Tuberculosis and were able to leverage patients diagnosed with COVID-19 via their participation in evaluations of new tuberculosis diagnostics (which typically require people with respiratory symptoms). Tuberculosis and COVID-19 have similar symptoms and risk factors, and this approach also permitted many participants from impoverished populations with high rates of human immunodeficiency virus (HIV) to be recruited. Another important source of participants at our medical campus were healthcare workers diagnosed with COVID-19 as part of occupational health screening.

*Integration of co-developed biorepositories*
 At both Johns Hopkins University (JHU) and the UMass Chan Medical School, co-existing biorepository efforts were underway simultaneously with the RADx TUB effort. JHU developed a very similar metadata collection tool that the teams were then able to align and fully integrate to allow the samples from JHU to be included in the TUB biorepository. The JHU biorepository also began recruitment in November 2020, before the TUB was launched, and therefore adds additional temporal diversity and variant representation. The JHU biorepository ultimately collected samples from 414 participants before full integration with TUB and an additional 109 participants (including 51 patients with COVID-19) under the common TUB protocol. UMass Chan worked closely with the UMass Memorial Health System to collect and retain remnant samples for thousands of patients who were treated in the UMass system. Although not characterized by the same metadata as the samples procured under the TUB protocol, this collection provides a large number of samples that add to the strength of our biorepository and can be accessed via the TUB. This collection includes 8,353 COVID positive nasal swabs, 10,682 COVID positive saliva samples, 216 COVID negative nasal swabs, and 192 COVID negative saliva samples
